# Cost-Effectiveness Evaluation of Bariatric Surgery for Morbidly Obese with Diabetes Patients in Thailand

**DOI:** 10.1155/2019/5383478

**Published:** 2019-02-03

**Authors:** Ithiphon Viratanapanu, Chavalit Romyen, Komol Chaivanijchaya, Sikarin Sornphiphatphong, Worawit Kattipatanapong, Ajjana Techagumpuch, Krit Kitisin, Suppa-ut Pungpapong, Chadin Tharavej, Patpong Navicharern, Patchaya Boonchayaanant, Suthep Udomsawaengsup

**Affiliations:** ^1^Department of Surgery, Faculty of Medicine, Chulalongkorn University, Bangkok, Thailand; ^2^Faculty of Pharmaceutical Science, Chulalongkorn University, Bangkok, Thailand; ^3^Department of Surgery, Faculty of Medicine, Thammasat University, Bangkok, Thailand; ^4^Department of Internal Medicine, Faculty of Medicine, Chulalongkorn University, Bangkok, Thailand

## Abstract

**Background:**

Bariatric surgery is a choice for treatment in morbidly obese patients with type 2 diabetes mellitus (DM type 2) who have inadequate diabetes control with only medical treatment. However, bariatric surgery requires highly sophisticated equipment, and thus the cost of surgery seems to be very high following the procedure compared with the cost of conventional diabetes care. This raises the question of whether bariatric surgery is cost-effective for morbidly obese people with diabetes in Thailand.

**Objective:**

To perform a cost-effectiveness evaluation of bariatric surgery compared with ordinary treatment for diabetes control in morbidly obese DM type 2 patients in Thailand.

**Methods:**

Cost-effectiveness study was conducted, using a combination of decision tree and Markov model in analysis. Treatment outcomes and healthcare costs were incurred by data from literature review and retrospective cohort in King Chulalongkorn Memorial Hospital from September 2009 to March 2016 for the conventional and bariatric surgery group, respectively. One-way sensitivity was used for analysis of the robustness of the model. Cost-effectiveness was assessed by calculating incremental cost-effectiveness ratios (ICERs). Monetary benefits at a threshold of 150,000 to 200,000 Thai baht (THB) per quality-adjusted life-year (QALY) based on the Thailand gross domestic products (GDP) value was regarded as cost-effectiveness of bariatric surgery.

**Results:**

Bariatric surgery significantly improves the clinical outcome including long-term diabetes remission rate, hemoglobin A1C, and body mass index (BMI). The incremental cost per QALY of bariatric surgery compared with the medication control is 26,907.76 THB/QALY which can consider bariatric surgery as a cost-effective option.

**Conclusions:**

Use of bariatric surgery in morbidly obese with DM type 2 patients is a cost-effective strategy in Thailand's context.

## 1. Introduction

Obesity is becoming a major global public health concern in every age group because many noncommunicable diseases are attributable to obesity. In the WHO 2016 report, an estimated proportion of more than 1.9 billion adults were overweight. Of these, over 650 million adults were obese. From 1991 to 2009, the prevalence of overweight and obesity in Thai population increased with an average of 0.95 kg/m^2^ per decade [1]. Recent data from Thailand in 2009 report prevalence of class I obesity and class II obesity was 26% and 9%, respectively [1].

Noncommunicable diseases such as cardiovascular disease (mainly heart disease and stroke), musculoskeletal disorders (especially osteoarthritis), and cancers are common health consequences of obesity. Diabetes mellitus (DM) is also one of the most important noncommunicable diseases related to obesity. Both diabetes and obesity are found to be the major risk factors that concomitantly increase the morbidity and mortality of patients.

American Diabetes Association (ADA) considered bariatric surgery as a choice of treatment for diabetes patients with BMI more than 35 kg/m^2^ [2]. Results of bariatric surgery combined with medical therapy were shown effectively more than medical therapy alone [2, 3].

Results of the recent systematic review suggest that the diabetic remission rate from bariatric surgery is about 60–80% over 1 year after the procedure and the probability of DM relapse will be reduced over time [4–7]. A large number of clinical trials have also demonstrated that bariatric surgery has a huge impact on glycemic control in obese patients with DM type 2, even achieving a long-term complete remission, a reduction in both microvascular and macrovascular complications, and an improvement in quality of life. Moreover, it reduces the utilization of the diabetes medicine leading to reduction in the long-term healthcare expense [3].

It can be estimated that obesity requires significant resources in the health sector for treatment and caring for patients with obesity-induced health problems. The economic costs of obesity included healthcare cost, cost of productivity loss due to premature mortality, and cost of productivity loss due to hospital-related absenteeism. Accounting for 0.13% of gross domestic products (GDP) was estimated as a total cost of obesity in Thailand, closely to the recent data from 10 Western European countries which estimated the cost of obesity to be as high as 0.09% to 0.61% of GDP [8].

Although the evidence suggests that bariatric surgery is a successful long-term treatment of obese with DM type 2 patients, it is an expensive procedure with additional costs possible in the months following surgery. This raises the question of whether bariatric surgery is cost-effective for severely obese people with diabetes.

There are several evidences of cost-effectiveness analysis of bariatric surgery for obese with DM type II patients among difference countries. One of the analysis from United States by Hoerger et al. [9] uses the Markov model to evaluate the bypass and banding surgery compared with usual diabetes care and found that surgery is either cost-effective or that leads to cost savings over time. This result is coherent with the work from United States by Wang et al. [10] that uses a two-part model and reports additional life expectancy of bariatric surgery compared with no surgery.

However, there are limited works on cost-effectiveness analysis of the bariatric surgery option in Thailand as people in our country might have the different context and threshold to accept the intervention. Therefore, we decided to conduct the cost-effectiveness study of bariatric surgery in morbidly obese diabetes patients in Thailand to evaluate this option in the systematic way.

## 2. Methodology

**Figure 1:**
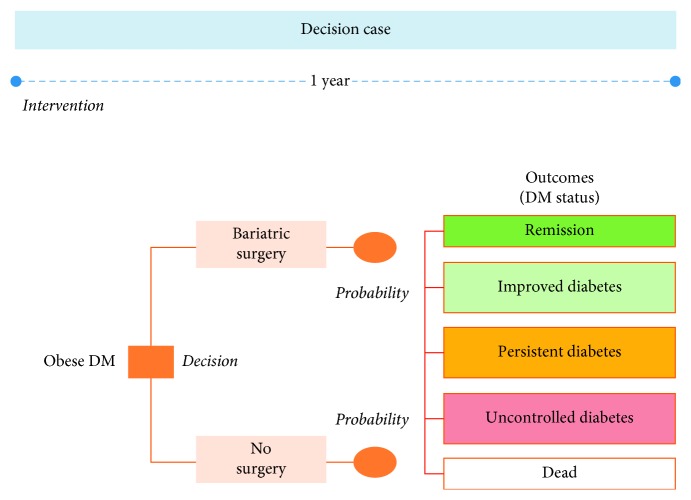
Decision tree model.

**Figure 2:**
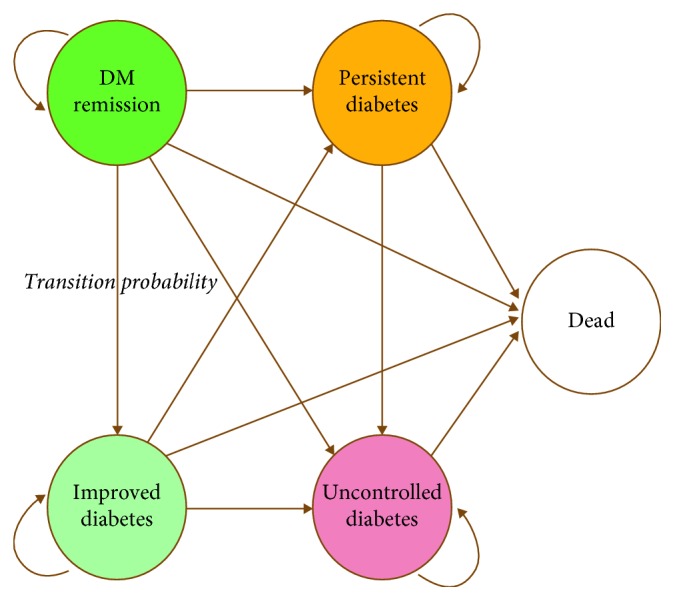
Markov model.

An outcome of cost-effectiveness evaluation study was conducted to analyze the cost-effectiveness of bariatric surgery compared with ordinary treatment for diabetes control in morbidly obese with DM type 2 patients, using a combination of a decision tree and a Markov model, as shown in Figures 1 and 2.

Treatment outcomes and healthcare costs incurred by data from literature review in the conventional group and retrospective cohort in King Chulalongkorn Memorial Hospital (KCMH) from September 2009 to March 2016 in the bariatric surgery group were modeled and compared between patients who underwent bariatric surgery and those who underwent conventional treatment for diabetes control. Inclusion criteria consist of DM type 2 patients with BMI more than 32.5 kg/m^2^ and had a follow-up time of more than 1 year.

Treatment outcomes were captured in terms of five diabetes status—diabetes remission, improved diabetes, persistent diabetes, uncontrolled diabetes, and dead which applied from ADA guidelines. Diabetes remission is defined as hemoglobin A1C (HbA1C) less than 6.5% without any diabetic-lowering agent. Improved diabetes is defined as HbA1C less than 6.5% with metformin monotherapy. Persistent diabetes is defined as the condition in which patients still use combination of metformin and another class of antidiabetic drug after treatment. Uncontrolled diabetes is defined as the condition in which patients use more than two types of antidiabetic drugs after treatment.

The decision tree illustrated the possible deterministic pathways of the two groups during the first year after surgery or initiation of medication. After the first year, the Markov model was used for diabetes treatment outcomes, with a time horizon of 50 years from the healthcare payer's perspective. In each annual cycle, a patient could move from each status outcome to the others or remained in the same state.

Data were collected at 6-month and 1-year periods which include the demographic data, types of bariatric surgery, clinical data including BMI and HbA1C, and utilization data for all diabetes status outcomes.

Healthcare costs were estimated for direct and indirect medical costs. Direct medical costs included bariatric surgery fee, diabetes medication fee, cost of supplementation, and cost of complication management. Indirect costs referred to productivity loss due to complication.

Cost valuation and measurement were based on the gross-costing method. A reference price (RP) is primary obtained from the explicit literature review, while utilization data are obtained from the KCMH database. In case of inadequate information, additional cost and utilization data will also be based on published literature.

The valuation of cost was based on an estimated cost incurred by patients treated at KCMH in each diabetes status outcome and Thai Food and Drug control Administration data. In diabetes remission, costs were assumed from one-time cost of bariatric surgery and annual cost of supplementation. In improved and persistent diabetes, costs were estimated from an incremental medication cost used for diabetes control. For uncontrolled diabetes, a total cost was accumulated from the cost of all antidiabetic drugs use and the cost of all complication management.

Effectiveness measurement included both short-term and long-term outcomes. For short-term outcomes including 1-year diabetes status, BMI, and HbA1C reduction, data were obtained from retrospective analysis. For long-term outcomes, data of probability of diabetes relapse, transition probability of the diabetes state, life-year gained, and utility were reviewed from published literature.

All costs and utility data were adjusted to 2017 price using Thailand Consumer Price Index (CPI) and presented in Thai baht (THB). All future costs were discounted at an annual rate of 3%.

Cost-effectiveness was assessed by the calculated incremental cost-effectiveness ratios (ICERs). ICERs in terms of cost per quality-adjusted life-year (QALY), cost per life-year gained, and cost per clinical benefits from BMI and HbA1C reduction were used in the model. The threshold for considering cost-effectiveness of bariatric surgery recommended by Thailand health technology acceptance guideline recommendation 2016 is accounted for one to three folds of gross domestic product (GDP) per capita which is around 200,000 THB per QALY [11, 12].

Sensitivity analysis was performed as the Tornado diagram and one-way deterministic sensitivity analysis (DSA) to examine the simultaneous uncertainty around each parameter including probability of diabetes remission and uncontrolled diabetes, cost of bariatric surgery and diabetes medication, annual discount rate, and utility of the diabetes status. In addition, a threshold analysis was used to evaluate the specified time to be the cost-effective bariatric surgery.

## 3. Results

From KCMH database, a total of 73 patients were included in the analysis. Of all, 43 (58.9%) patients were women, and mean (standard deviation, SD) age was 41.8 (12.2) years. Major type of surgery used in the KCMH setting was Roux-en-Y Gastric Bypass (RYGB) surgery that was conducted in 45 (61.6%) patients. Baseline characteristics of patients before receiving bariatric surgery in the KCMH database are shown in Table 1.

Healthcare costs for obese with DM type 2 patients from the KCMH database and literature review are shown in Table 2.

At 1 year after bariatric surgery compared with baseline, 61 (83.6%) patients had diabetes remission, mean (SD) BMI was 36.9 (8.9) kg/m^2^ vs. 50.9 (10.9) kg/m^2^ (*p* < 0.001), and mean (SD) HbA1C was 5.8 (1.4)% vs. 7.5 (1.9)% (*p* < 0.001). Clinical effectiveness data from the retrospective database analysis are shown in Table 3.

Probability of diabetes remission at 1 year after bariatric surgery was 0.8356. The probability of diabetes outcome between bariatric surgery and nonbariatric surgery group is shown in Table 4.

Utility data obtained from the literature review for each state of diabetes outcome are shown in Table 5.

Incremental cost per one unit decrease of HbA1C and BMI was 75,251.61 and 9,533.66 THB, respectively. Cost-effectiveness analyses between baseline and after bariatric surgery at the 1-year-period are shown in Table 6.

Discounted ICUR was 26,907.76 THB/QALY. Bariatric surgery is considered as a cost-effectiveness option for the Thailand healthcare setting as shown in Tables 7 and 8.

The result from threshold analysis suggested that bariatric surgery will start to be cost-effective at 7 years after the procedure.

**Figure 3:**
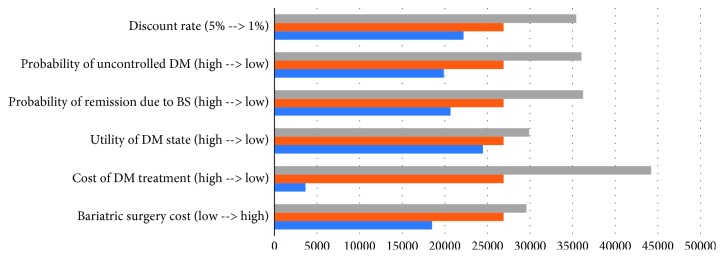
Result from one-way sensitivity analysis.

**Figure 4:**
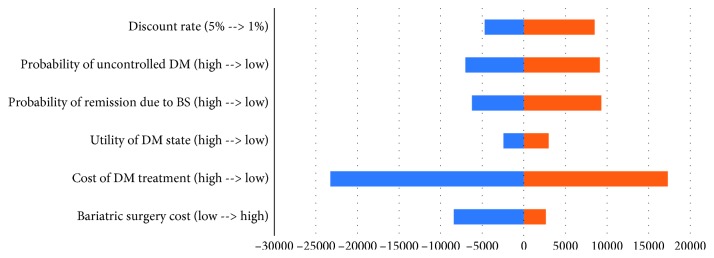
Tornado diagram.

The result from sensitivity analysis showed no change of discount rate, diabetes status, utility of the diabetes state, cost of DM treatment, and cost of bariatric surgery over the threshold, in which the cost of DM treatment has the highest impact on the model as shown in Figures 3 and 4.

## 4. Discussion

In our analysis, bariatric surgery was found to be a cost-effectiveness option based on incremental cost per QALY at around 27,000 THB which is below Thailand's threshold. The model is very robust based on deterministic sensitivity analysis that includes the change in parameter within the ninety percent interval.

The result found in our study was consistent with previous cost-effectiveness study of bariatric surgery in other countries. As different countries might have different contexts, bariatric surgery provided promising effectiveness including the control of diabetes without need of anti-diabetic medication and other meaningful benefit such as reduction in weight and the improvement in many clinical parameters.

Result from sensitivity analysis also suggests the robustness of the model. As we try to change the parameters including cost of bariatric surgery, cost of DM medication treatment, effectiveness of bariatric surgery, utility of DM stages, and discount rate to reflect the uncertainty of the result in real-world scenario, the incremental cost-effectiveness ratio is still below the Thailand threshold of cost-effectiveness.

The strength of our analysis is we including the data from both retrospective database analysis and explicit literature review. As the result of retrospective database analysis is gathered from the hospital database, it will present the data in local context very appropriately. However, as the lack of data in long-term follow-up and the lack of data in the control group, we need to use the data from the literature review instead of having different contexts with our desired target population.

In conclusion, the use of bariatric surgery in morbidly obese DM type 2 patients is considered as the cost-effectiveness strategy in Thailand's context.

## Figures and Tables

**Table 1:** Baseline characteristics of patients before receiving bariatric surgery in KCMH database (*N*=73).

Characteristics	Value
Female sex, *n* (%)	43 (58.9%)
Age, years^*∗*^	41.8 (12.2)
Baseline BMI (kg/m^2^)^*∗*^	50.1 (10.3)
Baseline body weight (kg)^*∗*^	135.8 (33.1)
Baseline HbA1C (%)^*∗*^	7.6 (1.9)
Baseline total cholesterol (mg/dL)^*∗*^	188.3 (48.4)
Baseline HDL (mg/dL)^*∗*^	43.2 (10.6)
Baseline LDL (mg/dL)^*∗*^	114.5 (41.9)
Baseline TG (mg/dL)^*∗*^	163.3 (98.7)
Baseline SBP (mmHg)^*∗*^	139.1 (18.8)
Baseline DBP (mmHg)^*∗*^	82.4 (12.0)

^*∗*^The values are expressed as mean (SD). BMI: body mass index; HbA1C: hemoglobin A1C; HDL: high density lipoprotein; LDL: low density lipoprotein; TG: triglyceride; SBP: systolic blood pressure; DBP: diastolic blood pressure; SD: standard deviation.

**Table 2:** Healthcare costs for morbidly obese DM type 2 patients.

Healthcare costs	Baseline value (range)^#^
Cost of bariatric surgery (THB/time)^*∗*^	150,000 (126,200–157,500)
Cost of DM medication (THB/year)^*∗∗*^	
1. Metformin	538.6 (269–3,331)
2. Sulfonylurea group	162 (154–168)
3. Thiazolidinedione group	12,840 (6,420–25,680)
4. Alpha-glucosidase inhibitors group	1,155.6 (577.8–1,964.4)
5. Insulin	3,480 (2,040–7,680)
Cost of vitamin and calcium supplementation (THB/year)^*∗∗*^	396 (367.2–568.8)
Cost of complications management (THB/years)^*∗∗∗*^	15,326.4 (3,672–25,895)

^*∗*^KCMH database; ^*∗∗*^National Drug System Development Committee Median Drug Price Announcement; Royal Gazette in Bangkok, Thailand, 2018; ^*∗∗∗*^reference [13]; ^#^the values are expressed as median; THB: Thai baht.

**Table 3:** Clinical effectiveness at 1 year after bariatric surgery.

Parameters	Before surgery	1 year after surgery	*p* value
BMI (kg/m^2^)^*∗*^	50.9 (10.9)	36.9 (8.9)	<0.001
HbA1C (%)^*∗*^	7.5 (1.9)	5.8 (1.4)	<0.001

^*∗*^Values are expressed as mean (SD).

**Table 4:** Diabetes outcome probability in both groups of morbidly obese DM type 2 patients.

Parameters	1 year after bariatric surgery^*∗*^	No bariatric surgery^*∗∗*^
Probability of diabetes remission	0.8356	0.0001
Probability of improved diabetes	0.0411	0.23
Probability of persistent diabetes	0.0099	0.17
Probability of uncontrolled diabetes	0.1233	0.5998
Probability of death	0.0001	0.0001

^*∗*^From KCMH database; ^*∗∗*^from Schauer, [14].

**Table 5:** Utility score after 1 year of bariatric surgery in each diabetes outcome of morbidly obese DM type 2 patients^*∗*^.

Utility score	Value
In diabetes remission	0.83
In improved diabetes	0.80
In persistent diabetes	0.78
In uncontrolled diabetes	0.75
In death state	0

^*∗*^Reference [15].

**Table 6:** One-year data of cost-effectiveness analysis compared between baseline and after bariatric surgery.

Type of outcomes	Incremental costs, THB	Incremental cost-effectiveness	ICERs
Incremental cost per HbA1C reduction	133,947.9	1.78	75,251.61
Incremental cost per BMI reduction	133,947.9	14.05	9,533.66

**Table 7:** Lifetime data estimated for cost-effectiveness analysis compared between bariatric surgery and nonbariatric surgery group among morbidly obese DM type 2 patients.

Parameters	BSG	Non-BSG	Incremental value (BSG vs. non-BSG)
Total discounted cost (THB)	436,928.72	360,810.89	76,117.83
Total discounted LYG (years)	17.45	14.15	3.30
Total discounted QALY (years)	13.57	10.75	2.83

BSG: bariatric surgery group; LYG: life-year gained; QALY: quality-adjusted life-year; THB: Thai baht.

**Table 8:** Incremental cost-effectiveness and incremental cost utility ratio.

Parameters	Value
Incremental cost (BSG vs. non-BSG)	76,117.83
Incremental life-year gain (LYG)	3.30
Discounted ICER	23,049.92
Incremental cost (BSG vs. non-BSG)	76,117.83
Incremental discounted QALY	2.83
Discounted ICUR	26,907.76

BSG: bariatric surgery group; ICER: incremental cost-effectiveness ratio; QALY: quality-adjusted life-year; ICUR: incremental cost utility ratio.

## Data Availability

The data used to support the findings of this study are available from the corresponding author upon request.
